# Radioimmunoimaging of Liver Metastases with PET Using a ^64^Cu-Labeled CEA Antibody in Transgenic Mice

**DOI:** 10.1371/journal.pone.0106921

**Published:** 2014-09-16

**Authors:** Stefanie Nittka, Marcel A. Krueger, John E. Shively, Hanne Boll, Marc A. Brockmann, Fabian Doyon, Bernd J. Pichler, Michael Neumaier

**Affiliations:** 1 Institute for Clinical Chemistry, Medical Faculty Mannheim, University of Heidelberg, Mannheim, Germany; 2 Department of Neuroradiology, Medical Faculty Mannheim, University of Heidelberg, Mannheim, Germany; 3 Department of Diagnostic and Interventional Neuroradiology, University Hospital of the Rheinisch-Westfaehlische Technical University Aachen, Aachen, Germany; 4 Department of Surgery, Medical Faculty Mannheim, University of Heidelberg, Mannheim, Germany; 5 Department of Preclinical Imaging and Radiopharmacy, Werner Siemens Imaging Center, University of Tuebingen, Tuebingen, Germany; 6 Department of Immunology, Beckman Research Institute, City of Hope, Duarte, California, United States of America; Baker IDI Heart and Diabetes Institute, Australia

## Abstract

**Purpose:**

Colorectal cancer is one of the most common forms of cancer, and the development of novel tools for detection and efficient treatment of metastases is needed. One promising approach is the use of radiolabeled antibodies for positron emission tomography (PET) imaging and radioimmunotherapy. Since carcinoembryonic antigen (CEA) is an important target in colorectal cancer, the CEA-specific M5A antibody has been extensively studied in subcutaneous xenograft models; however, the M5A antibody has not yet been tested in advanced models of liver metastases. The aim of this study was to investigate the ^64^Cu-DOTA-labeled M5A antibody using PET in mice bearing CEA-positive liver metastases.

**Procedures:**

Mice were injected intrasplenically with CEA-positive C15A.3 or CEA-negative MC38 cells and underwent micro-computed tomography (micro-CT) to monitor the development of liver metastases. After metastases were detected, PET/MRI scans were performed with ^64^Cu-DOTA-labeled M5A antibodies. H&E staining, immunohistology, and autoradiography were performed to confirm the micro-CT and PET/MRI findings.

**Results:**

PET/MRI showed that M5A uptake was highest in CEA-positive metastases. The %ID/cm^3^ (16.5%±6.3%) was significantly increased compared to healthy liver tissue (8.6%±0.9%) and to CEA-negative metastases (5.5%±0.6%). The tumor-to-liver ratio of C15A.3 metastases and healthy liver tissue was 1.9±0.7. Autoradiography and immunostaining confirmed the micro-CT and PET/MRI findings.

**Conclusion:**

We show here that the ^64^Cu-DOTA-labeled M5A antibody imaged by PET can detect CEA positive liver metastases and is therefore a potential tool for staging cancer, stratifying the patients or radioimmunotherapy.

## Introduction

Colorectal cancer is still one of the most common forms of cancer in Germany and the third most common cause of cancer-related deaths worldwide [1], [2]. An important target for the detection and monitoring of the recurrence of colon cancer is the human carcinoembryonic antigen (CEA, CEACAM5), a key member of the family of carcinoembryonic antigen-related cell adhesion molecules (CEACAMs) and a GPI-anchored cell surface glycoprotein that has been shown to be useful as a tumor-associated antigen and serum marker [3], [4]. The widely demonstrated overexpression of CEA in solid tumors can also be exploited to target tumor lesions by immunological methods [5] or for radioimmunotherapy (RIT) [6].

Radiolabeled antibodies have been frequently used in molecular imaging as PET tracers [7]–[10]. The fully humanized M5A variant of the murine T84.66 anti-CEA specific antibody can be efficiently labeled with ^64^Cu-DOTA [11]. Moreover, both M5A and T84.66 possesses a very high affinity for the CEA antigen (>10^10^ M^−1^) [12] with very low cross reactivity to other members of the CEACAM family and can be used as a whole antibody molecule.

Thus far, the evaluation of the ^64^Cu-labeled murine antibody T84.66 [13] or the fully humanized form M5A has been restricted to athymic nude mice bearing subcutaneous tumors [11]. In these studies, ^64^Cu-DOTA-M5A was capable of detecting xenograft tumors in nude mice. Our syngeneic orthotopic tumor model in the transgenic mouse strain C57BL/6 Han TgN (CEA-gen) allows us to study hematogenous liver metastases provoked by the intrasplenic injection of CEA-expressing colon tumor cells [14], [15]. These mice express CEA predominantly in the colon and intestine, with a spatial distribution of CEA comparable to that of human tissue [16], [17]. One major advantage of this syngeneic orthotopic mouse model is that the metastatic growth in this model, compared to subcutaneous tumor transplantation, is a more accurate model of the anatomic behavior, making this model highly attractive for the evaluation of novel imaging antibodies [18], [19].

Our aim was to evaluate the ^64^Cu-DOTA labeled M5A-antibody as a PET-tracer for imaging of liver metastases. Our approach was to inject C57BL/6-derived colon tumor cells into the spleens of syngeneic mice and then screen for metastases by micro-CT. As soon as lesions were detected, we proceeded with radioimmuno-PET/MRI with ^64^Cu-DOTA labeled M5A followed by histological confirmation of the imaging findings.

## Materials and Methods

### Ethical approval

All animal experiments have been conducted according to relevant national and international guidelines and were permitted by the Regierungspraesidium Karlsruhe and Regierungspraesidium Tuebingen and the Institutional Animal Care and Use Committees of the University Hospital Tuebingen and the University of Heidelberg.

### Cell lines

The murine cell line MC38, a syngeneic methyl-cholanthrene-induced colon cancer line [20], and the MC38-derivative cell line C15A.3 stably transfected with the CEACAM5 gene coding for human CEA [21], were used to induce a primary tumor in the spleen and hematogenic liver metastases. Both cell lines were grown in DMEM supplemented with 10% FCS, 4 mmol/L glutamine and penicillin/streptomycin (100 units/mL and 10 mg/mL). All media and reagents were bought from PAA (Pasching, Austria).

### Animals and tumor cell injections

C57BL/6 Han TgN (CEAgen) HvdP mice were generated as described previously [16]. Briefly, the cosmid clone cosCEAl, encompassing the complete human CEA gene including gene promoter regions sufficient for allowing tissue-specific gene expression [16], was used. Six-week-old female CEA-transgenic mice with a heterozygous CEA genotype were used for our studies. Mice were anesthetized, and 2*10^6^ C57BL/6-derived MC38 or C15A.3 cells in 50 µl PBS were injected into the spleen, giving rise to splenic tumors within 5–10 days post-injection. Prior to injection, CEA surface expression on C15A.3 cells using the murine T84.66 und humanized M5A CEA-specific monoclonal antibodies were tested by flow cytometry. C15A.3 cells were accepted for experiments if >95% were positive for CEA expression compared to MC38 and a mean fluorescence intensity above 80 or below 22, respectively. Mice were kept under standardized conditions and supplied with food and water *ad libitum* in a 12 h light-dark cycle.

### micro-CT Scans

For the time series-studies micro-CT scans were performed from days 9 to 19 and days 18 to 49 post-injection for MC38 or C15A.3 metastases, respectively. For all other experiments micro-CT imaging was performed at day 9 or 18 for the MC38 or C15A.3 cell lines, respectively, to detect liver metastases. If necessary, tumor-growth monitoring was repeated after another 7 days after the first pre-screening micro-CT, depending on the outcome of the first evaluation. Micro-CT was performed as described recently [22], [23]. Briefly, micro-CT imaging was performed using an industrial X-ray inspection system (Y.Fox; Yxlon International GmbH, Hamburg, Germany) equipped with a transmission X-ray tube and a 12-bit direct digital flatbed detector (PaxScan 2520; Varian, Palo Alto, CA, USA). A single dose of 100 µl of a liver-specific nanoparticulate contrast agent (VISCOVER ExiTron nano 6000; MiltenyiBiotec, Bergisch-Gladbach, Germany) was injected intravenously 3 hours prior to the first micro-CT scan. Mice were anesthetized, relaxed (Rocuronium, Esmeron, EssexPharma, München), and intubated before liver imaging was performed during a single breath stop within a 40 sec scan time during continuous image acquisition at 30 fps (frames per second) using the following scan parameters: 80 kV; 75 mA; and 180° rotation [22], [23]. Relaxation afterwards was reversed by the *intra peritoneal* (*i.p.*) injection of 20 mg/kg body weight of sugammadex (BridionH; EssexPharma, Munich, Germany). The acquired projections were reconstructed using a filtered back-projection algorithm with a 512×512×512 matrix using the software Reconstruction Studio (Yxlon International GmbH, Hamburg, Germany). Analysis of reconstructed images was performed using the public domain software OsiriX (v3.5.1; www.osirix-viewer.com).

### ^64^Cu Labeling of monoclonal M5A antibody

Humanized hu14.18 monoclonal anti-GD2 antibody as control was conjugated to commercially available NHS-DOTA (Macrocyclics, Dallas, TX, USA) as described previously [10]. The number of chelate molecules per immunoglobulin was not determined. The fully humanized DOTA labeled M5A monoclonal anti-CEA antibody was kindly supplied by John E. Shively (Beckman Research Institute of City of Hope, Duarte, CA) and NHS-DOTA conjugation was performed as described previously. The number of chelate molecules per immunoglobulin was determined to be ∼8 as analyzed by MALDI-TOF [11]. The antibody was produced in accordance to GMP-standards through a service available to collaborators with City of Hope (J.E.S.). The purified antibody-DOTA conjugates were labeled with ^64^Cu as described previously [24]. Briefly, ^64^Cu was produced in Tuebingen by irradiating ^64^Ni at a 16 MeV cyclotron (GE Healthcare, Uppsala, Sweden) and isolating it from ^64^Ni and other metals by using ion exchange chromatography [25]. Radiolabeling of DOTA-M5A and DOTA-hu14.18 was performed by adding ∼20 MBq of ^64^CuCl_2_ buffered in 10xPBS to ∼20 µg of antibody in PBS and incubation for 1 h at 42°C. pH was checked to be at 7.0. Quality control was performed by thin-layer chromatography on Polygram SIL G/UV_254 nm_ (Machery-Nagel, Dueren, Germany) plates and analyzed on a Cyclone Plus phosphor imager (Perkin Elmer, Waltham, Massachusetts, USA). Only antibody preparations with a labeling efficiency of >90% were used for experiments.

### PET- and MR Imaging

Approximately 2–4 days after the first detection of liver metastases by micro-CT imaging, animals were given a tail vein injection of ∼13 MBq of ^64^Cu-labeled anti-CEA mAb M5A-DOTA or anti-GD2 mAb hu14.18-DOTA (∼20 µg). For the blocking experiments, 500 µg of unlabeled M5A antibody was injected 3 h prior to the injection of ^64^Cu-labeled M5A antibody.

Ten-minute static PET scans were obtained at 3, 24 and 48 h after tracer injection on an Inveon dedicated small animal PET scanner (Siemens Preclinical Solutions, Knoxville, Tennessee, USA). Animals were anesthetized with 1.5% isoflurane (Abbott, Wiesbaden, Germany) evaporated in oxygen at a flow of 0.5 L/min, and body temperature was maintained at 37°C by a heating pad and a rectal temperature sensor. Images were reconstructed with an iterative ordered-subset expectation maximization algorithm. According to our standard protocol for mouse PET imaging, attenuation and scatter correction were not applied. Images were reconstructed in Inveon Acquisition Workplace 1.5.0.28 with OSEM2D with four iterations. The reconstructed voxel size was 0.776×0.776×0.796 mm. After each PET scan, the animals were transferred to a 7 T ClinScan MR scanner (Bruker, Ettlingen, Germany), and anatomic images were acquired with a 3D turbo-spin-echo (tse) sequence (TE = 205 ms, TR = 3000 ms, voxel size: 0.22×0.22×0.22 mm^3^, matrix size: 160×256×120).

### Analysis of PET and MR images

PET and MR images were overlaid and analyzed in Inveon Research Workplace (Siemens Preclinical Solutions, Erlangen, Germany) by rigid fusion. Images were overlaid by manually overlaying three markers that were filled with ^64^Cu dissolved in PBS and placed in proximity to the animals during the measurements. Liver lesions, primary tumors and other organs were identified and regions of interest (ROI) were drawn in the MR images. Volumes of interest (VOI) were calculated from these ROI's in Inveon Research Workplace and lesion volumes and %ID/cm^3^ were determined over the whole lesion volume.

### Determination of ^64^Cu-DOTA-M5A blood half-life

For determination of blood half-life, ∼13 MBq of ^64^Cu-labeled anti-CEA mAb M5A-DOTA (∼20 µg) were injected in mice bearing C15A.3 metastases and PET images were acquired at 3; 24 and 48 h post injection. Volumes of interest were drawn in the right ventricle of the animals and the %ID/cm^3^ was determined. A single exponential fit was used to calculate the blood half-life.

### Immunohistochemistry for Paraffin sections

Mice were killed immediately after the last 48 h PET/MR imaging and major organs were removed. For some animals organs were fixed in 4% of PBS buffered formalin prior to tissue processing and 3–5 µm thick serial paraffin sections were prepared for immunohistochemistry or routine H&E staining.

For immunohistochemistry, sections were deparaffinized and subjected to antigen retrieval by high temperature technique in 1 mM EDTA-Tris buffer (pH 9.0, 30 minutes at 270 W in a microwave oven). Sections were allowed to cool down to room temperature. After incubation in PBS/0.5% Tween20 for 5 minutes, endogenous peroxidase was blocked by immersion in 3% H_2_O_2_/Methanol for 20 minutes. The non-specific binding was blocked by incubation with 5% normal goat serum/2% BSA-PBS for 30 minutes at 37°C. M5A primary antibody was diluted to 2.0 µg/mL in 0.1% BSA-PBS and incubated for 45 minutes at 37°C. Sections were washed with water and incubated in PBS for 5 minutes. This was followed by incubation with the diluted secondary reagent goat anti-human-HRP antibody (1/500; Dianova, Hamburg, Germany) for 45 minutes at 37°C. After an additional washing step, antibody binding was visualized by adding diaminobenzidine and subsequent hematoxylin counterstaining.

Routine H&E staining of at least one section adjacent to slides subjected to immunostaining was performed to assess tissue differentiation as well as position and number of metastases.

All slides were evaluated by microscopy (Diaplan, Leica, Nussloch, Germany) and microphotographs were taken.

### Immunochemistry with cryosections and autoradiography

In some cases after the 24 h PET/MRI scan, the liver was immersed in TissueTek (Sakura, Alphen aan den Rijn, Netherlands) and frozen at −20°C and 7 µm or 20 µm thick cryosections were prepared in an alternating fashion to compare adjacent slices in immunohistochemistry, H&E and autoradiography. 20 µm thick frozen sections were subjected to autoradiography and routine H&E staining and 7 µm serial sections were used for immunohistochemistry. A total of 10 sections were prepared for each liver.

For immunohistochemistry with frozen sections, frozen slices were subjected to endogenous peroxidase block using 0.3% H_2_O_2_/Methanol for 20 minutes, followed by immunostaining steps as described for paraffin sections.

For autoradiography frozen slices were fixed on SuperFrost microscope slides (R. Langenbrick, Emmendingen, Germany) and placed in a shielded cassette (Amersham, Glattbrugg, Switzerland) where a phosphoscreen (Amersham) was exposed to the samples for 24 h. Subsequently the screens were analyzed on a Storm 840 Phospho Imager (Amersham) with a resolution of 50 µm.

All slides were evaluated by microscopy (Diaplan) and microphotographs were taken.

### Statistical Analysis

For statistical analysis an ANOVA with a Fisher LSD test was performed. Values were considered to be statistically significantly different at *P* values of 0.05 or lower.

Quantitative values are reported as mean +/− one standard deviation.

## Results

### Tumor cells and development of liver metastases

**Figure 1:**
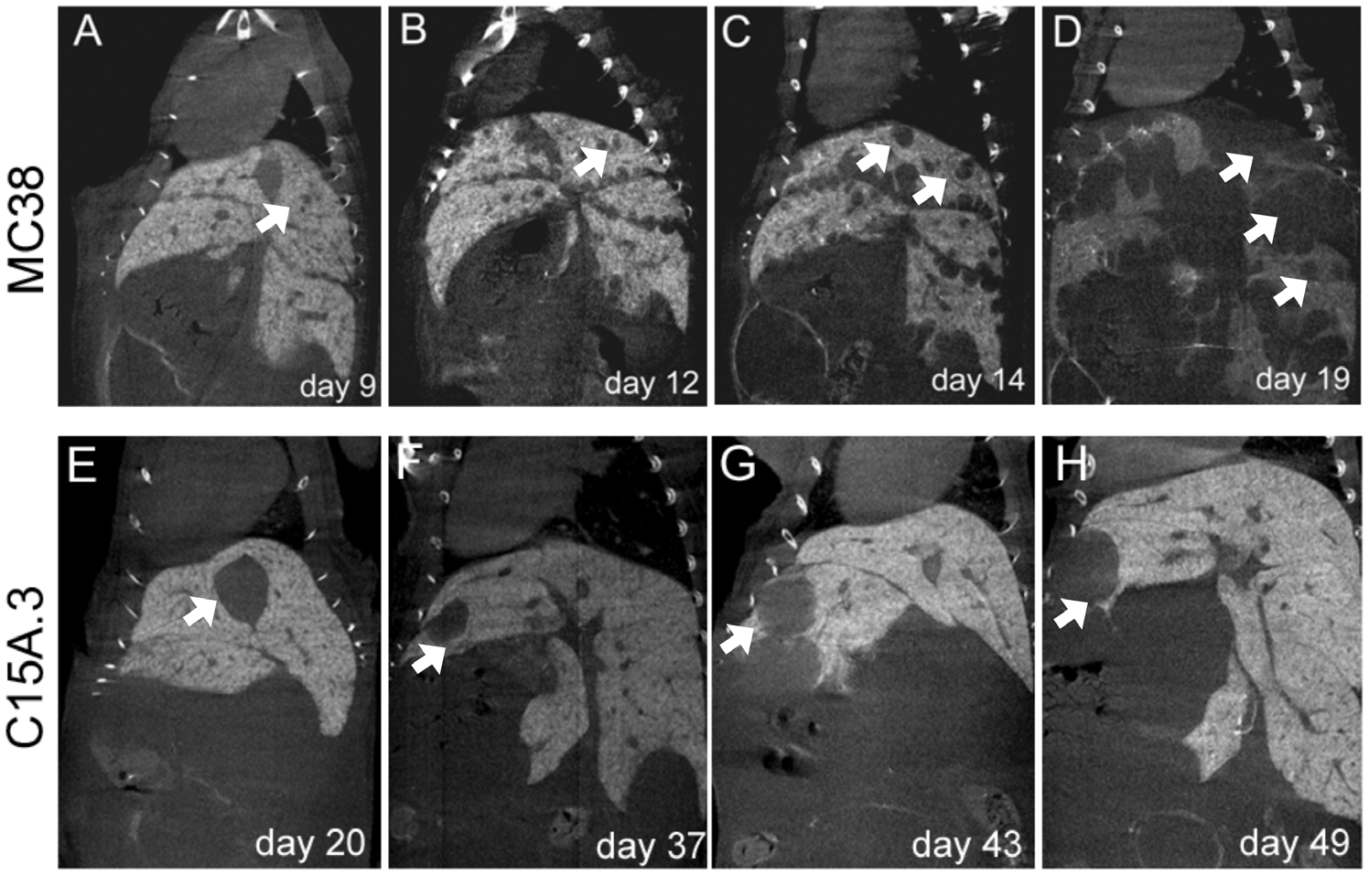
The development of liver metastases in mice monitored by micro-CT. To establish a suitable pre-screening routine, a separate group of animals was subjected to a time-series of micro-CT scans. Images were acquired 3 h post injection of a single dose of hepatocyte-specific contrast agent (ExiTron nano 6000) without the use of additional injections for follow-up. Images were collected at days 9 to 19 and days 20 to 49 for CEA-negative MC38 or CEA-positive C15A.3 metastases, respectively. Arrows indicate the same metastases at different time points for both cell lines. Note the differences in the size and number of metastases originating from MC38 (upper row) compared to C15A.3 (lower row).

CEA transgenic mice were injected with either CEA-expressing (C15A.3) or CEA-negative (MC38) cells and subsequently micro-CT scans were performed as a pre-screening method. Typically, 9–16 days post-injection, the first metastases were present in the mice injected with MC38 cells, whereas metastases originating from the C15A.3 cells were usually detectable between days 18–25. Micro-CT imaging with ExiTron nano 6000 allowed observation of the development of metastases as demonstrated in Figure 1 over a time course of 31 days. Lesions as small as 0.9 mm in diameter could easily be identified as dark round structures (Figure 1). For all animals, primary tumors were detected in the spleen, ranging from 0.9 mm to 12.0 mm in diameter. No visual correlation was found between the size of the primary tumor and the size or number of metastases.

### Immunohistological characterization of liver metastases

To further characterize our tumor model and to independently confirm the findings in micro-CT, we performed histological staining of the liver and other organs. At least 3 sections of each organ were evaluated by standard H&E staining, but metastases were found exclusively in the liver of the animals. The metastases had substantial size and diameter variability. As already suspected on the basis of the micro-CT scans, metastases originating from MC38 tumor cells were smaller but more frequent than those derived from C15A.3 tumor cells.

Tissue sections from the liver and spleen were immunostained using the M5A monoclonal antibody, and CEA-immunoreactivity could be demonstrated for all C15A.3 metastases but not for the MC38 CEA-negative metastases.

**Figure 2:**
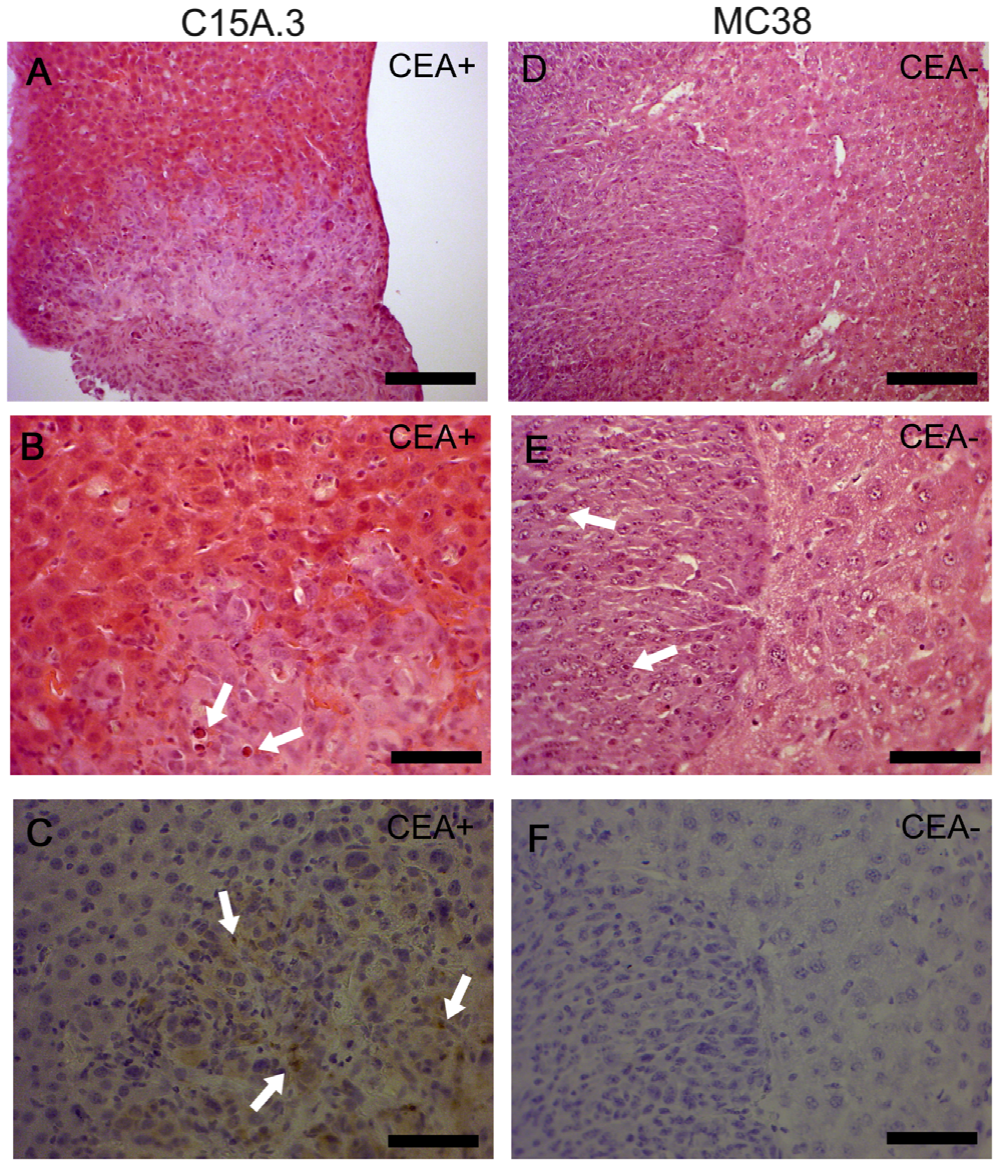
Liver histopathology of metastases derived from CEA-positive C15A.3 murine tumor cells (A–C) or CEA-negative parental MC38 colon tumor cells (D–F). (A, D) H&E staining of liver metastases surrounded by normal tissue. (B, E) Higher magnification, emphasizing the difference in the tumor cell growth patterns with respect to the normal-to-tumor tissue border. (C, F) Immunohistochemistry of serial sections stained for CEA with CEA-specific M5A antibody confirming the CEA-expression status of the metastases. Note many central necrotic cells as indicated by arrows in B, C and E in both metastases. Bar size (A, D) 125 µm, all other 50 µm.

The staining patterns for C15A.3 liver metastases appeared to be heterogeneous. The central areas of larger liver metastases were sometimes interspersed with necrotic cells, which are easily recognized by their small condensed or fragmented nuclei (Figure 2).

### Detection of liver metastases by ^64^Cu-labeled M5A-DOTA and *in vivo* biodistribution

Despite the fact that ^64^Cu-DOTA-labeled antibodies are known to show high nonspecific background in liver tissue [10], the excellent binding properties of the CEA-specific M5A antibody reported in previous studies [11] encouraged us to test this antibody for the detection of CEA-expressing liver lesions.

**Figure 3:**
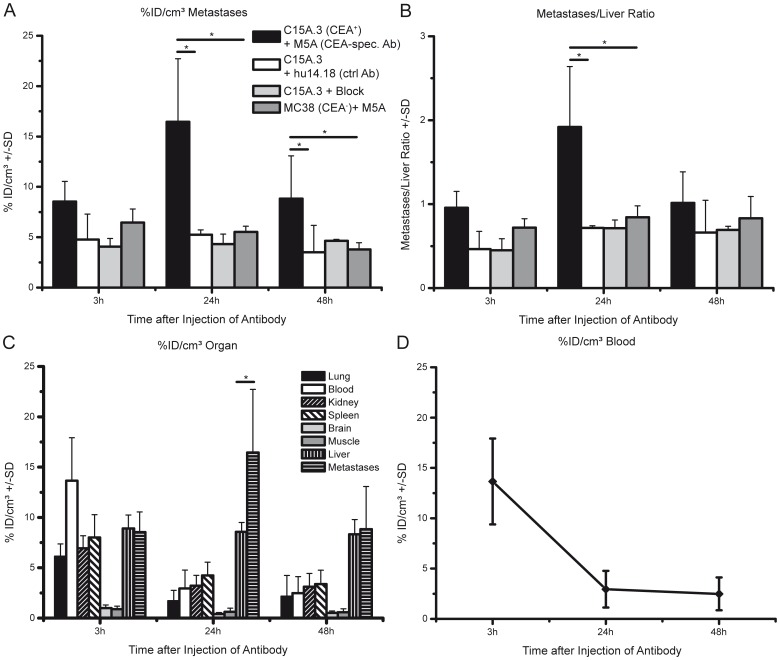
Uptake of the ^64^Cu-DOTA-labeled M5A antibody in CEA-positive C15A.3- and CEA-negative MC38-derived liver metastases. (A) Depicted are the %ID/cc of the labeled M5A antibody in either C15A.3 or MC38 derived metastases and the uptake of the control antibody hu14.18 at three different time points post injection of antibodies. For blocking experiments, 500 µg of unlabeled M5A was injected 3 h prior to labeled M5A. (B) The ratio of the antibody uptake between metastases and healthy liver. For A+B n = 8 animals for C15A.3+M5A, n = 7 animals for MC38+M5A, n = 2 animals for blocking and n = 4 animals for control antibody (hu14.18). All animals were scanned at every time-point. (C) %ID/cm^3^ of ^64^Cu-DOTA-labeled M5A antibody in several organs at different time points. (D) Focus on the %ID/cm^3^ of ^64^Cu-DOTA-labeled M5A in the right ventricle. For C+D n = 7 at all time points. Statistically significant results are designated with * when p≤0.05.

To test the M5A antibody in our model, we injected ^64^Cu-DOTA-labeled M5A into mice, which were identified as positive for liver metastases by micro-CT within 3 days. PET/MRI studies were performed at three time-points (3; 24 and 48 h) after antibody injection. Liver lesions were easily detected by MRI at all time points and ranged from 1 mm to 13 mm. Antibody uptake was visible at 3 hours post injection but sufficient uptake in liver metastases was reached after 24 h of tracer uptake. The %ID/cm^3^ in CEA-expressing C15A.3 metastases at 24 h post injection was 16.5% ±6.3% and was significantly higher than in healthy liver tissue in the same animals (8.6%±0.9%) and CEA-negative MC38 metastases in control animals (6.0%±0.8%; p≤0.05) (Figure 3). Somewhat unexpectedly, the uptake decreased after the 24 h time point.

**Figure 4:**
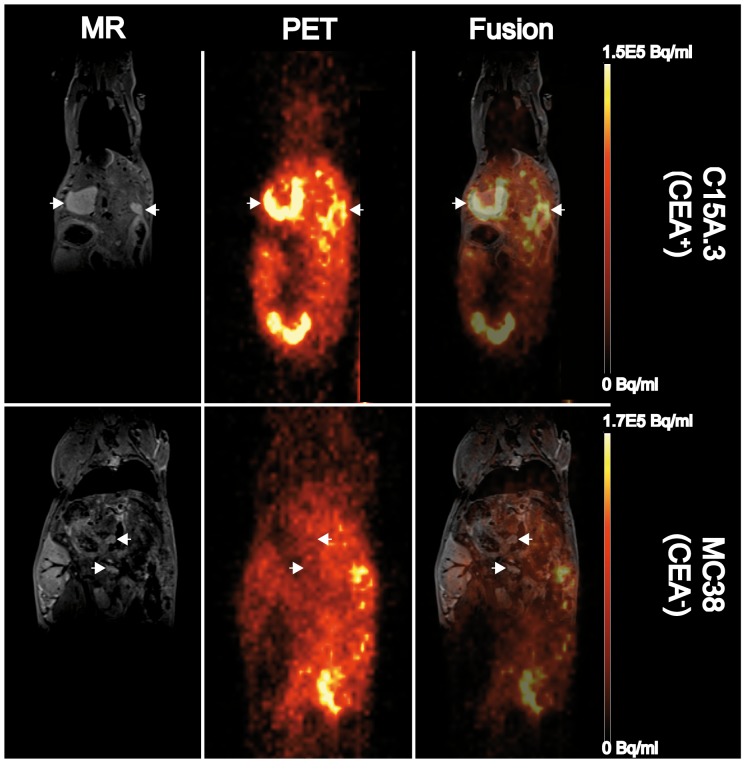
MRI and PET scan results for liver metastases originating from C15A.3 and MC38 cells 24 h post-injection of ^64^Cu-DOTA-M5A antibody. MRI images clearly show the location of the liver metastases (arrows) and were used to draw regions of interest (ROIs) for evaluating the PET data. Immuno-PET images indicate strong signals in the areas of the CEA-positive C15A.3-derived liver metastases. No enhanced tracer uptake was observed in the areas of CEA-negative MC38 derived metastases.

The ratio of the %ID/cm^3^ between the C15A.3-derived metastases and healthy liver tissue was 1.9±0.7, while the ratio of the MC38 metastases and healthy liver tissue was significantly lower (0.9±0.2; p≤0.05). Thus, we could visually discriminate between healthy liver tissue and CEA-positive lesions (Figure 4).

Additionally in all animals injected with C15A.3 cells and the M5A antibody, several organs were analyzed for M5A uptake in *in vivo* whole body PET images at the three time-points. We could clearly see that in all organs analyzed, except liver and metastases, the uptake decreased over time. In liver the %ID/cm^3^ stayed relatively stable over the time analyzed (Figure 3).

To determine the blood half-life time of the ^64^Cu-DOTA-labeled M5A antibody, the %ID/cm^3^ in the right ventricle was determined 3; 24 and 48 h after antibody injection and a blood half-life of 18.7 h was calculated.

### Verification of M5A specificity

To prove the specific binding of the M5A antibody in CEA-expressing metastases and to exclude nonspecific enhanced perfusion and retention (EPR) effects, we repeated these experiments with the fully humanized GD2-specific hu14.18 antibody, which should not bind to C15A.3 tumors and metastases. Thus, the GD2-specific hu14.18 antibody signal represents the level of nonspecific background binding of antibodies in this tissue. We did not observe a significant increase in the %ID/cm^3^ over time for the control antibody in C15A.3 lesions, but we did detect a significantly lower uptake of the control antibody in the C15A.3-derived liver metastases after 24 h of tracer injection (5.3%±0.5% ID/cm^3^) compared to M5A (Figure 3).

Additionally, we tested M5A specificity with blocking experiments. We injected mice bearing C15A.3 liver metastases with unlabeled M5A antibody 3 h prior to the injection of the ^64^Cu-DOTA-labeled M5A antibody. The large amount of unlabeled M5A was expected to bind most of the available specific binding sites within the metastases, decreasing the enrichment of the labeled M5A. PET and MRI scans were performed at different time points post injection of the labeled M5A. There was a significant reduction in the uptake of the M5A antibody into the C15A.3 metastases and tumor tissue from 16.5±6.3%ID/cm^3^ to 4.3±1.0%ID/cm^3^ at 24 h post injection of ^64^Cu-DOTA-M5A (Figure 3). These results clearly show that the high uptake of M5A after 24 h in the C15A.3 metastases is not due to nonspecific tissue-related effects.

### Correlation of the histological findings and PET imaging results

Closer examination of the PET imaging results showed central lesion regions with low tracer-related signals for some of the larger liver metastases (Figure 4). This finding was in agreement with our histological findings described earlier but was still subjected to intensive *ex vivo* examination.

For this purpose, animals were killed immediately after the 24 h PET/MR scan, which showed a peak in M5A uptake. The livers were removed, and autoradiography, H&E staining and immunochemistry were performed on cryosections of the liver.

**Figure 5:**
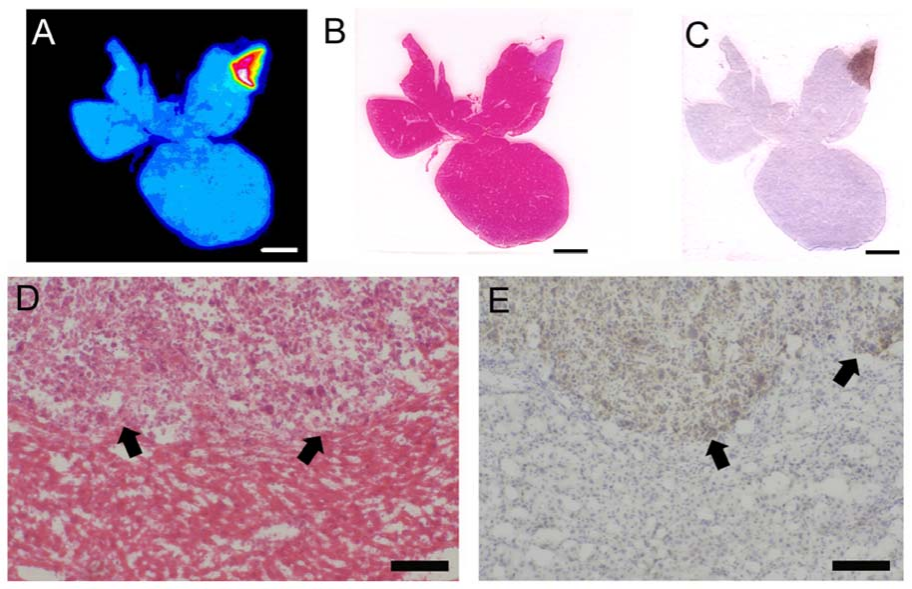
*Ex vivo* autoradiography (A), H&E staining (B, D) and M5A-immunohistochemistry (C, E) of the CEA-positive C15A.3-derived metastases. Evaluation of the cryosections was performed 24 h post-injection of ^64^Cu-DOTA-M5A antibody. (A) Autoradiography shows the heterogeneous distribution of M5A within metastases. (B, C) The location of metastases in H&E and immunostaining correlates with the location of the metastases shown in (A). Note the intense, dark staining of the C15A.3 tumor cells near the tumor-to-normal border (D, E arrows). Bar size A-C 3 mm and C-D 200 µm respectively.

In general, the autoradiography findings confirmed the heterogeneous antibody distribution in the large liver metastases, as seen in *in vivo* PET scans. We also noted a pronounced immunohistochemical signal at the border of the larger metastases, indicating higher antigen expression or antibody binding in these areas. H&E staining revealed necrotic areas in the center of large lesions (Figure 5). In general, areas of high CEA expression as seen by immunochemistry correlated with areas of high signal in autoradiography, whereas areas of necrosis shown by H&E correlated inversely with the autoradiography signal.

## Discussion

It has been previously reported that the M5A antibody shows outstanding binding characteristics in subcutaneous CEA tumor models [11]. Therefore, we decided to use this promising antibody in a more challenging and novel model of syngeneic liver metastases.

Obviously, when testing a tumor-specific PET probe for the screening or monitoring of liver lesions *in vivo*, it is necessary to select a suitable time point for the beginning of the immunoimaging. Therefore, we performed micro-CT scans to confirm the presence of liver lesions in the animals used for further immunoimaging studies. To confirm CEA expression in our model of liver metastases, we performed immunostainings with M5A after the final micro-CT or PET/MRI-scan. These experiments showed CEA expression in all C15A.3-derived liver metastases, supporting the usefulness of our syngeneic tumor model in testing novel CEA-targeting PET tracers.

The ^64^Cu-DOTA-M5A antibody in our tumor model showed high M5A uptake in CEA-positive C15A.3 lesions 24 h after antibody injection, while uptake in CEA-negative MC38 lesions stayed at a background level. The uptake in C15A.3 lesions was lower than in subcutaneous LS-174T tumors as reported by Li et al. [11]. The differences in the cell type and tumor location could both contribute to a different tumor microenvironment, explaining the discrepancy in the results.

We are aware of the fact that the ^64^Cu-DOTA complex is unstable *in vivo* and release of ^64^Cu will lead to enhanced uptake in healthy liver, which is the organ of interest in this study [10], [26]. Other chelators like NOTA, crossbridged cyclams, and others [27]–[30] have been shown to give better tumor to liver ratios *in vivo*, but since the M5A antibody has already been labeled with DOTA under GMP conditions and clinical studies have been performed with ^64^Cu-DOTA-M5A, we still decided to use DOTA in this work to increase the clinical relevance of this study. Therefore it is especially notable that the lesion-to-liver-ratio of tracer uptake for C15A.3 lesions allowed clear identification of lesions in PET/MR-images. Although for clinical studies a higher lesion-to-liver ratio would be appreciated, we still believe that for diagnostic purposes a lesion-to-liver ratio of ∼2 is for most cases sufficient for stratification. Alternatively for future studies other chelators could be used. For RIT it would be important to perform similar biodistribution studies with therapeutic isotopes like ^177^Lu or ^90^Y, since the high unspecific background observed here is probably to large parts due to free ^64^Cu released from the DOTA complex. Therefore a translation of the observed lesion-to-liver ratios to other isotopes is speculative, since the complex stability of these isotopes with DOTA is different from ^64^Cu.

Interestingly, the lesion-to-liver ratio observed here with the full M5A antibody outcompetes the ratios reached with a ^64^Cu-DOTA labeled T84.66 derived 80 kDa minibody (T84.66 is the parental antibody of M5A) [31], although smaller antibody formats are commonly expected to have better pharmacokinetic properties when used as imaging probes [32]. One possible explanation for this might be the higher affinity of the M5A antibody compared to the minibody (1.1×10^10^ vs.2–3×10^9^M^−1^ respectively) [33], [34].

Although both cell lines used were based on the same cellular background (MC38 or MC38 stably expressing human CEA), we could see differences in the size, number and growth rate of liver metastases by micro-CT, histology and PET/MRI. MC38 cells generated smaller but more frequent metastases that appeared more quickly than C15A.3-derived metastases (data not shown). This result is comparable with findings by Hand et al. [35], who showed reduced growth rates of subcutaneous tumors positive for human CEA in C57BL/6 mice.

The differences in the size and number of metastases made us question whether MC38-derived tumors or metastases are a suitable control for our purposes because antibody uptake in tumors is well known to be strongly affected by factors such as tumor size, vascularization or interstitial pressure [36]. Since the amount of vascularization, interstitial pressure and other factors were not determined for the liver metastases of either origin and the tumor size can affect the antibody uptake due to partial volume effects, necrotic areas or decreased perfusion in larger tumors, we also injected the GD_2_-specific hu14.18 antibody into animals bearing C15A.3 lesions, which are GD_2_-negative. Since the uptake of this control antibody stayed at a background level, we were able to exclude the possibility of high M5A uptake in C15A.3 lesions due to physiological reasons. Furthermore, blocking experiments were performed, showing that M5A uptake can be decreased by pre-injecting unlabeled M5A, again showing that the high uptake of M5A in C15A.3 lesions does not depend on nonspecific differences in tumor physiology, indicating the specificity of M5A.

In several PET images, we could clearly identify a heterogeneous distribution of ^64^Cu-DOTA-M5A in C15A.3 lesions, especially within larger liver metastases. When this phenomenon occurred, the rims of the lesions showed high signal, and the cores showed low signal. These findings could be supported by autoradiographies of livers performed after PET/MRI scans. In some cases, the M5A immunostaining of the same livers used for the autoradiographies showed increased CEA expression in the rims, correlating with the findings in PET and autoradiography. Furthermore, H&E staining of the autoradiography sections revealed necrotic areas in the centers of the metastases, which might be poorly perfused. Therefore, both effects might account for the heterogeneous tracer distribution within liver metastases. Additionally, these observations could be attributed to the high affinity of M5A [33], which could lead to a rapid sequestration of the antibody to the antigen and interfere with the free diffusion of M5A into the lesions, thus preventing antibody uptake into the tumor core. This effect is known as a ‘binding-site barrier’ effect, which was first described by Fujimori et al. [37].

Since the ROIs in this study were drawn based on the MR images and no necrosis was visible in the MR images, lesion areas with low PET signal were included in the %ID/cm^3^, making the high M5A-uptake for C15A.3 metastases even more impressive.

Finally, we observed a wash-out effect of M5A between the 24 h and 48 h time point. Our results stand in contrast to data generated by Li et al. [11] for subcutaneous LS-174T tumors in which there was an increase in antibody uptake over the first 48 h upon injection. However, this discrepancy might again be due to the differences in the tumor models used.

## Conclusions

The binding properties of M5A were analyzed for the first time in a syngeneic orthotopic model of liver metastases that matches the scenario for human patients much more closely than common subcutaneous tumor models. The outstanding binding properties of the M5A antibody enabled us to generate signal-to-noise ratios in the liver that were high enough to clearly identify CEA-expressing liver metastases. This is the first report showing the characterization of liver lesions with a ^64^Cu-DOTA-labeled antibody despite the high nonspecific background in healthy liver tissue. Therefore, this study proposes that the M5A antibody, which is currently undergoing clinical Phase I/II RIT trials [6], could be a useful tool to diagnose CEA-positive liver metastases for patient stratification and subsequent RIT.
